# Online racism, online mental health engagement, depression, and suicide risk among Asian American and Pacific Islander and Black American emerging adults

**DOI:** 10.3389/fpubh.2026.1745978

**Published:** 2026-02-26

**Authors:** Brian TaeHyuk Keum, Feng Wang, Leona M. Ofei, Michele J. Wong, Hans Oh, Vanessa V. Volpe

**Affiliations:** 1 Department of Community Health Sciences, University of California, Berkeley, Berkeley, CA, United States; 2 Lynch School of Education and Human Development, Boston College, Chestnut Hill, MA, United States; 3 Department of Community Health Sciences, University of California, Los Angeles, Los Angeles, CA, United States; 4 Asian American Research Center, Institute for the Study of Societal Issues, University of California, Berkeley, Berkeley, CA, United States; 5 School of Social Work, University of Southern California, Los Angeles, CA, United States; 6 Department of Psychology, North Carolina State University, Raleigh, NC, United States

**Keywords:** AAPI, Black, depression, online racism, social media, suicide

## Abstract

**Introduction:**

Recent studies suggest that exposure to racist online interactions and content may be associated with depressive symptoms and suicide risk among racially minoritized individuals. Despite the hate and violence easily encountered online, individuals use social media platforms to engage with their mental health concerns and seek support. Hence, the current study aimed to explore whether online mental health engagement would buffer the depressive symptoms and suicide risk associated with online racism among Black and Asian American Pacific Islander (AAPI) emerging adults (18–29) who are exposed to anti-Black and anti-Asian hate online.

**Method:**

Using survey data from 1,224 Black (*M*_age_ = 23.99, *SD* = 3.11) and 1,553 AAPI (*M*_age_ = 23.96, *SD* = 3.29) emerging adults, we conducted latent moderated structural equation modeling to examine online mental health engagement as a moderator between online racism and depressive symptoms, and online racism and suicide outcomes [suicide ideation in the past 2 weeks, lifetime suicide ideation, past-year suicide ideation, lifetime suicide attempt, past-year suicide attempt, and non-suicidal self-injury (NSSI)].

**Results:**

For both groups, online mental health engagement associated with a stronger link between online racism and suicide-related risk. For AAPI emerging adults, higher levels of engagement significantly amplified the association between online racism and suicidal ideation (recent and lifetime), suicide attempts (lifetime), NSSI, and depressive symptoms. For Black emerging adults, this risk-exacerbating finding was observed only for recent suicidal ideation.

**Discussion:**

The findings highlight the suicide risk that may be associated with online racism and concerns regarding the helpfulness of online mental health engagement.

## Introduction

Racist content and harassment are pervasive online and disproportionately affect minoritized racial/ethnic groups, particularly Asian American and Pacific Islander (AAPI) and Black American emerging adults ages 18–29 (1). According to the Pew Research Center, anti-Asian discrimination has increased since the COVID-19 pandemic, including threats, physical attacks, being called offensive names, and being treated as foreigners. There has also been an uptick in anti-Black violence and discrimination in the wake of the murder of George Floyd (2). Online racism is operationally defined as a multifaceted phenomenon, encompassing overt hate speech, subtle bias, direct victimization, and vicarious trauma (3). In many online spaces, such as social media platforms and gaming chats, direct and vicarious forms of racism and harassment are widespread (3). When engaging in these online settings, an individual may encounter racial slurs or racially charged threats directed at them or vicariously witness racist or violent content (e.g., police brutality) targeting their racial group or other marginalized populations (4). Black and AAPI emerging adults have reported poor mental health from experiencing these online racism experiences, including depressive symptoms and suicide ideation (5, 6). Crucially, while online racism precipitates psychological distress, emerging adults already experiencing high levels of suicide ideation may increase their time spent in digital spaces as a coping mechanism, thereby increasing their probability of encountering further racist victimization.

### Online racism and suicide risk

The disproportionate burden of online racism among AAPI and Black American emerging adults could significantly elevate their risk for suicide. Building upon prior evidence showing adverse mental health consequences tied to online racism among racially minoritized emerging adults (5, 7, 8), recent studies have begun to highlight the link between online racism and suicide ideation among racially minoritized emerging adults (5, 6, 9, 10). For instance, in a racially diverse sample of undergraduate college students (ages 18–30 years), online racial/ethnic discrimination was directly associated with increased suicide ideation and alcohol misuse (10). A key theoretical mechanism driving this link may be digital re-exposure (3), where the viral and permanent nature of online content exposes individuals to repeatedly witness and relive traumatic racial events, compounding the psychological burden.

Research among AAPI and Black American emerging adults highlights interpersonal risk factors as requiring further attention when examining how online racism could increase their risk for suicide (5). For example, in a study employing the interpersonal theory of suicide [IPTS; (11)], Keum (5) found that perceived burdensomeness but not thwarted belongingness mediated the link between online racism and suicide ideation among Black, AAPI, and Latinx groups, with this pathway being fully mediated among the AAPI group. The findings indicated that online racism may be associated with suicide ideation primarily by fostering a sense of being a liability to one's community rather than by eroding social ties. Specifically, thwarted belongingness—defined as the painful psychological state where an individual's fundamental need for social connection and belonging is unmet—did not serve as the driving factor in this model. Since data collection occurred in 2021, the authors noted that the context of pandemic-related anti-Asian sentiment may have pushed AAPI emerging adults who encounter online racism to feel like more of a burden to society. Building on these findings, it is important to examine ways that can counter the socially marginalizing interpersonal risk factors (e.g., feeling like a burden) to mitigate the impact of online racism on suicide risk.

### Social media engagement on mental health

In the digital era, one timely and digitally-relevant approach to counter the interpersonal risk associated with online racism may be people's engagement and connection to social media communities regarding mental health concerns. In this study, online mental health engagement is operationally defined as the active or passive interaction with digital content related to mental health, including seeking information, participating in peer-support forums, or following mental health-focused accounts. Research has found that emerging adults utilize internet and social media sources for online mental health engagement (12–14), but findings are mixed on the extent to which they are helpful or harmful for mental health help-seeking. For instance, online mental health engagement offers space for emerging adults to learn more about mental health and connect with others with similar experiences while enabling anonymity (12, 15, 16). Functionally, online mental health engagement likely serves as a digital extension of social support, fulfilling similar roles by providing informational resources, emotional validation through shared experiences, and instrumental guidance for those who face barriers to traditional care.

At the same time, some scholars suggest the quality of mental health information on the internet needs improvement (17, 18), and findings are inconsistent on whether these sources improve or exacerbate mental health stigma (19, 20). Furthermore, exposure to mental health information from the internet or social media sources can influence mental health decision making including self-diagnosis, medication management, and the encouragement of self-harm and pro-suicide attitudes (21–24). These findings call for additional clarification on the extent to which, and for whom, these sources promote help-seeking or treatment use and whether this ultimately addresses symptom reduction. This is particularly pertinent for Black and AAPI emerging adults who indicate some of the lowest rates of treatment use (25, 26), despite accelerating rates of suicide outcomes (27–29).

Black and AAPI emerging adults report using the internet and social media for mental health engagement (14, 30, 31). Yet, internet and social media exposure also put Black and AAPI emerging adults at risk for increased online racism exposure (31, 32). However, limited research has examined whether engagement in online mental health tools and resources limits or exacerbates the negative mental health impact of online racism for these emerging adults. This is important to consider given the double-edged nature of online mental health engagement highlighted previously. The role of online mental health engagement as a moderator may be either protective or risky depending on the underlying psychological mechanism. As a protective mechanism, online mental health engagement may buffer the impact of racism through collective coping and racial socialization, allowing individuals to externalize the source of their distress. Conversely, it may function as a risk factor through implications such as emotional contagion (33), where the constant exposure to others' distress intensifies one's own negative affect, a process often exacerbated by algorithmic reinforcement loops that prioritize increasingly distressing content.

Furthermore, studies of offline discrimination suggest that mental health resources such as social support may not always reduce the negative impact of racism on mental health and psychological distress for Black and AAPI individuals (34–36). Therefore, it is possible that the potential social support that online mental health engagement may offer may not be enough to limit the impact of online racism on mental health. This is substantiated by research that finds that while social support lessens the impact of vicarious online racism on anxiety, it loses its buffering capacity for direct online racism. Furthermore, social support had no moderating effect on the impact of both vicarious and direct racism on depressive symptoms (37). It is possible that the varied symptomatology for various mental health outcomes (e.g., fear and vigilance for anxiety vs. hopelessness and anhedonia for depression) (38, 39) could mean that mental health resources work differently for various outcomes. These findings warrant the investigation of specific outcomes, such as suicide, to identify the extent to which online mental health engagement may uniquely limit vs. amplify the impact of online racism on suicide for Black and AAPI emerging adults.

### The present study

Based on our review, we examined whether online mental health engagement would moderate the link between online racism and depressive symptoms and suicide outcomes. Given that research to date documents both benefits and harms of engaging in social media, our hypotheses were exploratory. On one hand, online mental health engagement may buffer the link between online racism and suicide risk/depressive symptoms for those who may be able to find online support for their mental health issues. On the other hand, the linkages could be exacerbated for those who are not able to find effective or unhelpful support that could expose them to scrutiny, shame, and further isolation. Below were our hypotheses for both Black and AAPI groups:

- Online racism would significantly predict depressive symptoms and suicide risk (Lifetime suicide ideation, past-year suicide ideation, recent suicide ideation, lifetime suicide attempt, past-year suicide attempt, and self-harm behavior).
- Online mental health engagement would significantly moderate the above links. Our examination of the moderation was exploratory, given that literature documents both benefits and harms.

## Method

### Participants and procedure

This study is based on cross-sectional data collected between July and September 2024 from 1,224 Black and 1,553 Asian American/Native Hawaiian/Pacific Islander emerging adults in the U.S., aged 18–29. This study was approved by the University of Southern California IRB (APP-24-05114). Participants were recruited via quota-based convenience sampling from a Qualtrics survey panel, which consists of pre-recruited individuals from various online sources who have agreed to take part in research. To ensure the quality of the online sample and that participants were human, several data integrity checks were performed. These included an initial CAPTCHA screening and multiple proprietary verification procedures used by Qualtrics' panel partners, such as digital fingerprinting and the validation of demographic and address information. Responses from outside the United States were removed, along with responses that originated from the same IP address. Two attention checks were also included. All participants provided informed consent at the start of the survey and were given incentives for their completion. Inclusion criteria required participants to be between 18–29 years old, identify as Black and/or AAPI, even if they also identified with another race (e.g., White, Middle Eastern, Native American, Latino), reside in the United States, and be able to read English. Individuals who selected multiple racial categories were categorized as “Multiracial Black or Multiracial AAPI.” Only individuals who completed 100% of the survey were included in the final recruitment quotas. Overall, 15,876 individuals started the survey, with 2,800 providing complete responses, resulting in a completion rate of 17.6%. Table 1 provides a comprehensive overview of the core study variables and demographic covariates by racial groups.

**Table 1:** Descriptive statistics of suicide-related outcomes by racial group.

**Variable**	**Black American (*N* = 1,224)**	**AAPI (*N* = 1,543)**
**Core study variables**		
PORS	2.59 (1.09)	2.68 (1.13)
OMHE	2.22 (1.13)	2.23 (1.28)
Lifetime SI	33.5%	33.7%
Past-year SI	*N* = 410; 60.0%	*N* = 520; 57.1%
Lifetime attempt	19.7%	19.6%
Past-year attempt	*N* = 241; 39.4%	*N* = 302; 56.3%
NSSI	14.4%	18.5%
Recent SI (past 2 weeks)	0.83 (1.04)	0.97 (1.11)
Depressive symptoms	10.12 (7.13)	11.26 (7.53)
**Demographic covariates**		
techfreq	3.40 (0.85)	3.46 (0.73)
gender: Man	49.6%	46.0%
gender: Woman	50.4%	54.0%
sex: Male	50.6%	47.7%
sex: Female	49.4%	52.3%
orientation: Heterosexual	82.6%	82.5%
orientation: Homosexual	4.5%	4.2%
orientation: Bisexual	10.3%	9.5%

Values are means (standard deviations) for continuous variables and percentages for dichotomous variables. PORS, Perceived Online Racism Scale (4); OMHE, Online Mental Health Engagement; Lifetime SI, Ever seriously thought about suicide; Past-year SI, Serious suicidal ideation within the past 12 months; Lifetime Attempt , Ever attempted suicide; Past-year Attempt, Suicide attempt in the past 12 months; NSSI, Non-suicidal self-injury in the past month; Recent SI, Suicidal ideation in the past 2 weeks; Depressive symptoms, PHQ-9 total score; techfreq, Daily internet use frequency, measured by how often participants go online for any purpose (40). Sample sizes varied across variables due to skip patterns.

### Measures

#### Online racism

Frequency of exposure to online racism in the past 6 months was measured using the Perceived Online Racism Scale - Very Brief (41). This is a six-item scale with a 5-point Likert response scale from Never to Always. Items included the following: “Seen other racial/ethnic minority users receive racist comments,” “Encountered online hate groups/communities against non-white racial/ethnic groups,” “Seen photos that portray my racial/ethnic group negatively,” “Been informed about a viral/trending racist event that I was not aware of,” “Seen online videos (e.g., YouTube) that portray my racial/ethnic group negatively,” “Received posts with racist comments.” A total score was calculated based on the sum of the Likert scale values for all items. Higher scores reflect greater exposure to online racism.

#### Depression

Severity of depression was assessed using the 9-item Patient Health Questionnaire (PHQ-9). The PHQ-9 is a widely used and validated instrument (42, 43) demonstrating high internal consistency (α = 0.91) and moderate to moderately high correlations (0.52–0.85) with clinical diagnoses. Participants self-rated the frequency of depressive symptoms (e.g., “Little interest or pleasure in doing things”) over the past 2 weeks on a 0–3 Likert scale (“Not at all” to “Nearly every day”). Total scores (1–27) correspond to depression severity levels, with scores ≥10 indicating moderate-to-severe depression for this analysis.

#### Online mental health engagement

Participants indicated how often they had engaged in mental health communications online in the past year using four items. The items included: (1) join forums or closed social media groups on specific mental health topics, (2) do hashtag searches for mental health topics on social media, (3) follow people online who share about mental health conditions, and (4) share about your own poor mental wellbeing online. Participants indicated the frequency of each item using a Likert-type response scale from 0 (Never) to 4 (Once a week or multiple times a week). A mean score was computed from items to represent the overall frequency of online mental health communication, such that a higher score indicates more frequent online mental health communication. The internal consistency of the scale was acceptable (α = 0.87).

#### Suicide ideation and attempt

Suicidal ideation and behaviors were assessed using five dichotomous items adapted from the National Survey on Drug Use and Health (44). Lifetime suicide ideation was measured by asking participants whether they had ever seriously thought about suicide, with a follow-up item assessing whether such thoughts occurred within the past 12 months. Similarly, suicide attempts were assessed via questions about lifetime attempts and attempts in the past year, with the latter asked only if a previous attempt was reported. Non-suicidal self-injury (NSSI) behavior in the past month (NSSI) was measured by asking participants whether they had ever intentionally injured themselves without wanting to die. All responses were coded as 1 (Yes) or 0 (No), and each outcome was analyzed separately to capture distinct temporal dimensions of suicide-related risk.

#### Time spent on the internet

Participants were asked the following: “On a typical day, how often do you use the internet?” Responses were on a 5-point scale from Almost constantly to Not at all. This question was adapted from the 2022 California Health Interview Survey (40).

#### Sociodemographic characteristics

Age (Range:18–29), Sex assigned at birth (Male, Female), Sexual orientation (Heterosexual, Homosexual (Gay/Lesbian), Bisexual, Not listed), Education [(Less than a high school diploma), (High school, GED, some college or technical training), (Graduate, college, or technical degree)], Nativity (Born in the U.S, Not born in the U.S), Food insecurity (Yes vs. No), Region of the country (Midwest, Northeast, South, West).

### Analysis

Before testing the hypothesized interaction effects, we first conducted a confirmatory factor analysis (CFA) using the lavaan package in R to evaluate the measurement structure of our latent constructs. The latent variable of online racism was modeled as being indicated by six items of the PORS-VB, each assessing distinct yet related aspects of digital racial hostility or exposure. The latent construct of online mental health engagement was defined by four items reflecting participants' frequency of engagement with online mental health content, such as joining mental health forums or sharing their own experiences on social media. CFA results indicated excellent model fit: χ^2^(34) = 410.63, *p* < 0.001, CFI = 0.979, TLI = 0.973, RMSEA = 0.063, and SRMR = 0.030. All item factor loadings were statistically significant (*p* < 0.001), with standardized values ranging from 0.762 to 0.823 for online racism and from 0.760 to 0.869 for online mental health engagement, demonstrating robust latent variable representation. Following the CFA, we split the dataset by racial group into two subgroups: Black American (*N* = 1,225) and AAPI (*N* = 1,543) emerging adults. This subgrouping allowed us to examine whether the relationships among online racism, mental health engagement, and suicide-related outcomes operated similarly across these distinct populations, given their differing cultural experiences, social media environments, and mental health stigma contexts.

To test whether the relationship between online racism and suicide-related outcomes varied as a function of individuals' engagement in digital mental health communication, we conducted Latent Moderated Structural Equation Modeling (LMS) using Mplus 8 (45). The LMS approach was originally developed to estimate interactions between latent variables within a maximum likelihood framework and has been widely used in psychological and behavioral research (46–48). LMS assumes that latent variables are continuous and approximately normally distributed, and that model identification follows standard constraints for latent interaction models (46). Given the large sample size and the use of well-established multi-item indicators, these assumptions were considered reasonable for the present analyses, consistent with prior applications of LMS in behavioral and mental health research (48). This approach allows for the direct estimation of multiplicative latent interactions without requiring the construction of product indicators, making it particularly suited for this study as both the predictor (online racism) and the moderator (online mental health engagement) were specified as latent variables.

A total of seven suicide-related outcomes were examined separately as dependent variables, including past 2 weeks' suicidal ideation (item 9 of the PHQ-9), lifetime and past-year suicidal ideation, lifetime and past-year suicide attempts, past-month NSSI, and depressive symptoms (latent PHQ-9), which were modeled as a latent construct indicated by nine items of the PHQ-9.

Notably, the analytic sample size varied across outcomes due to differential item presentation. Among Black American participants, sample sizes were *N* = 1,224 for lifetime suicidal ideation and suicide attempts, *N* = 410 for past-year suicidal ideation, and *N* = 242 for past-year suicide attempts. Among AAPI participants, sample sizes were *N* = 1,543 for lifetime suicidal ideation and suicide attempts, *N* = 520 for past-year suicide ideation, and *N* = 300 for past-year suicide attempts. These differences reflect skip patterns in the survey, whereby past-year outcomes were only asked of individuals who endorsed corresponding lifetime experiences. To account for unequal effective sample sizes across outcomes and potential deviations from normality associated with these skip patterns, all LMS models were estimated using robust maximum likelihood estimation (ESTIMATOR = MLR), which provides standard errors and test statistics robust to non-normality (45).

For each outcome, we estimated a structural model that included online racism, online mental health engagement, and their latent interaction (specified via the XWITH command in Mplus) as predictors, while controlling for relevant covariates: sex assigned at birth, gender identity, sexual orientation, and daily technology use frequency. We controlled for sex assigned at birth and gender identity, as research indicates that the prevalence and qualitative nature of online harassment often differ across the gender spectrum (4). Furthermore, sexual orientation was included as a covariate to account for intersecting minority stressors that may independently compound psychological distress and suicide risk for AAPI and Black emerging adults. Finally, we controlled for daily technology use frequency to ensure that the relationship between online racism and mental health outcomes is not simply a byproduct of a higher “dosage” of internet exposure or a general propensity for high-frequency digital engagement. A total of 14 models were estimated separately within the AAPI and Black American subsamples to allow group-specific inferences regarding the psychological impact of online racism and the potential amplifying or buffering role of online mental health engagement. Given that the moderation hypothesis was tested repeatedly across multiple suicide-related outcomes, we applied false discovery rate (FDR) correction using the Benjamini–Hochberg procedure to the interaction terms (PORS × OMHE) within each racial group in order to control for Type I error inflation (49). Main effects and covariates were not adjusted, as they were not part of the repeated hypothesis-testing family.

To interpret latent interaction effects, we examined conditional effects using simple slope analyses and predicted values, an approach recommended for probing interactions in latent moderated structural equation models, in Mplus (45, 46). For both continuous and binary outcomes, simple slopes were evaluated at low (−1 SD), mean, and high (+1 SD) levels of the moderator. For continuous outcomes [e.g., Recent suicidal ideation (past 2 weeks)], we plotted predicted values across standardized levels of online racism at three levels of the moderator (−1 SD, 0, +1SD), using unstandardized regression coefficients obtained from latent moderated structural equation models. All x-axes were standardized to range from −2 to +2, capturing the majority of the observed range of the latent predictor. For binary outcomes (e.g., Lifetime SI), conditional effects were examined by transforming model-estimated logits into predicted probabilities using the logistic function, allowing for an interpretable visualization of interaction patterns under a non-linear link function. All figures were generated in R using the ggplot2 package, with color and line type used to represent levels of online mental health engagement.

## Results

### Measurement model

To assess the measurement properties of the two primary latent constructs—online racism and online mental health engagement—we conducted a confirmatory factor analysis (CFA) using the lavaan package in R. Each construct was modeled as a unidimensional latent factor, with online racism indicated by six items capturing distinct yet converging forms of digital racial hostility, and online mental health engagement indicated by four items (mhion1–mhion4) assessing participants' engagement with mental health-related content online. The CFA demonstrated excellent fit to the data, χ^2^(34) = 410.63, CFI = 0.979, TLI = 0.973, RMSEA = 0.063, SRMR = 0.030. All factor loadings were significant (*p* < 0.001), with standardized values consistently above 0.75 for both constructs, indicating strong convergent validity. To further evaluate the reliability and convergent validity of the latent factors, composite reliability (CR) and average variance extracted (AVE) were computed based on the standardized CFA solution. Results indicated high internal consistency for both online racism (CR = 0.91, AVE = 0.63) and online mental health engagement (CR = 0.90, AVE = 0.69), exceeding commonly recommended thresholds for acceptable reliability and convergent validity. The latent correlation between online racism and online mental health engagement was moderate (*r* = 0.40), suggesting related but distinct constructs and indicating that multicollinearity was unlikely to bias parameter estimates in subsequent latent interaction models. Given these robust psychometric properties, the latent factors were retained for use in subsequent structural models. All analyses were conducted separately for AAPI and Black American participants to examine group-specific patterns.

### Main effects and covariates

We first examined the main effects of online racism and online mental health engagement on suicide-related outcomes across the two racial groups. Structural models were estimated separately for Black American and AAPI emerging adults, with each of the seven outcomes modeled individually. Across both groups, online racism was consistently and positively associated with multiple suicide-related indicators, including suicidal ideation for the past 2 weeks, lifetime suicidal ideation past-month NSSI behavior, and depressive symptoms. Online mental health engagement similarly showed positive associations with several outcomes, although it's predictive strength and consistency varied by group.

For Black American participants (*N* = 1,225), perceived online racism was significantly associated with increased risk for three out of five suicide-related binary outcomes (see Table 2). Specifically, higher levels of online racism were associated with higher odds of lifetime suicidal ideation [OR = 1.614, 95%CI (1.386, 1.879), *p* < 0.001], lifetime suicide attempts [OR = 1.419, 95%CI (1.196, 1.683), *p* = 0.001], and NSSI [OR = 1.533, 95%CI (1.262, 1.863), *p* < 0.001]. However, its associations with past-year suicidal ideation [*N* = 410, OR = 1.011, 95%CI (0.771, 1.324), *p* = 0.940] and past-year suicide attempts [*N* = 242, OR = 1.251, 95%CI (0.869, 1.800), *p* = 0.280] were not statistically significant. In addition, online racism was positively associated with suicidal ideation (SI) in the past 2 weeks [*B* = 0.284, 95%CI (0.210, 0.359), *p* < 0.001], and with elevated depressive symptoms [*B* = 0.224, 95%CI (0.166, 0.281), *p* < 0.001; see Table 3].

**Table 2:** Odds ratios of logistic regression results predicting mental health and suicide-related outcomes by predictor variables (Black American sample).

**Outcome variable**	**PORS**	**OMHE**	**PORS X OMHE**	**techfreq**	**Gender**	**Sex**	**Orientation**
Lifetime SI	1.614^***^ [1.386, 1.879]	1.087 [0.932, 1.268]	0.94 [0.825, 1.070]	0.991 [0.851, 1.154]	1.416 [1.042, 1.925]	0.885 [0.589, 1.329]	1.240^***^ [1.121, 1.372]
Past-year SI	1.011 [0.771, 1.324]	1.353 [1.012, 1.809]	0.814 [0.632, 1.048]	0.991 [0.774, 1.269]	1.085 [0.815, 1.444]	0.542^**^ [0.323, 0.910]	0.996 [0.878, 1.129]
Lifetime Attempt	1.419^**^ [1.196, 1.683]	1.197 [1.009, 1.419]	0.991 [0.860, 1.143]	1.12 [0.941, 1.332]	1.043 [0.702, 1.549]	1.348 [0.825, 2.202]	1.125^*^ [1.030, 1.229]
Past-year attempt	1.251 [0.869, 1.800]	1.407 [0.938, 2.109]	0.788 [0.578, 1.075]	0.772 [0.535, 1.113]	0.96 [0.697, 1.323]	0.496^**^ [0.249, 0.989]	0.788 [0.554, 1.122]
NSSI	1.533^***^ [1.262, 1.863]	1.376^**^ [1.147, 1.649]	1.041 [0.898, 1.208]	0.945 [0.790, 1.131]	1.113 [0.728, 1.703]	0.939 [0.549, 1.608]	1.130^*^ [1.018, 1.255]

Values are odds ratios (OR).

^*^p < 0.05, ^**^p < 0.01, ^***^p < 0.001.

*P*-values for the interaction terms (PORS × OMHE) were adjusted for multiple comparisons using the FDR procedure within the Black American sample. PORS, Perceived Online Racism Scale (4); OMHE, Online Mental Health Engagement; PORS × OMHE, Interaction term between perceived online racism and online mental health engagement; techfreq, Daily internet use frequency, measured by how often participants go online for any purpose (40); gender, Gender identity as self-reported (1 = Man, 2 = Woman, 3 = Other); sex, Sex assigned at birth (1 = Male, 2 = Female, 3 = Intersex); orientation, Sexual orientation (1 = Heterosexual, 2 = Homosexual, 3 = Bisexual, 4 = Other). Outcome variables: Lifetime SI, Ever seriously thought about suicide; Past-year SI, Serious suicidal ideation within the past 12 months; Lifetime Attempt, Ever attempted suicide; Past-year Attempt, Suicide attempt in the past 12 months; NSSI, Non-suicidal self-injury in the past month. Sample sizes varied across outcomes due to skip patterns; estimates for past-year outcomes are based on smaller subsamples and should be interpreted with caution.

**Table 3:** Coefficients of linear regression results predicting mental health and suicide-related outcomes by predictor variables (Black American sample).

**Outcome variable**	**PORS**	**OMHE**	**PORS X OMHE**	**techfreq**	**Gender**	**Sex**	**Orientation**
Recent SI (past 2 weeks)	0.284^***^ [0.210, 0.359]	0.208^***^ [0.139, 0.276]	0.086^**^ [0.027, 0.145]	−0.070^*^ [−0.133, −0.007]	0.127^*^ [0.022, 0.232]	−0.172^*^ [−0.323, −0.021]	0.045^*^ [0, 0.090]
Depressive symptoms	0.224^***^ [0.166, 0.281]	0.156^***^ [0.101, 0.212]	0.026 [−0.022, 0.073]	−0.006 [−0.051, 0.039]	0.103 [−0.005, 0.211]	−0.023 [−0.156, 0.110]	0.047^*^ [0.009, 0.086]

Values are coefficients (B).

^*^p < 0.05, ^**^p < 0.01, ^***^p < 0.001.

*P*-values for the interaction terms (PORS × OMHE) were adjusted for multiple comparisons using the FDR procedure within the Black American sample. Recent SI, Suicidal ideation in the past 2 weeks; Depressive symptoms, PHQ-9 total score; PORS, Perceived Online Racism Scale (4); OMHE, Online Mental Health Information Engagement; PORS × OMHE, Interaction term between perceived online racism and online mental health engagement.

Online mental health engagement was positively associated with recent suicidal ideation [*B* = 0.208, 95%CI (0.139, 0.276), *p* < 0.001], depressive symptoms [*B* = 0.156, 95%CI (0.101, 0.212), *p* < 0.001], and NSSI [OR = 1.376, 95%CI (1.147, 1.649), *p* = 0.003]. It was marginally associated with past year suicidal ideation [*N* = 410, OR = 1.353, 95%CI (1.012, 1.809), *p* = 0.078], but not with lifetime suicidal ideation [OR = 1.087, 95%CI (0.932, 1.268), *p* = 0.310], lifetime suicide attempts [OR = 1.197, 95%CI (1.009, 1.419), *p* = 0.059], or past-year suicide attempts [*N* = 242, OR = 1.407, 95%CI (0.938, 2.109), *p* = 0.162].

Among AAPI participants (*N* = 1,543), both online racism and online mental health engagement showed significant associations with several suicide-related outcomes (see Tables 4, 5). Online racism was a robust predictor across outcomes, with significantly higher odds observed for lifetime suicidal ideation [OR = 1.710, 95%CI (1.388, 2.106), *p* < 0.001], lifetime suicide attempts [OR = 1.980, 95%CI (1.444, 2.715), *p* = 0.002], and NSSI [OR = 1.808, 95%CI (1.316, 2.484), *p* = 0.006]. It was also associated with higher recent (past 2 weeks) suicidal ideation [*B* = 0.338, 95%CI (0.255, 0.421), *p* < 0.001] and greater depressive symptoms [*B* = 0.337, 95%CI (0.276, 0.398), *p* < 0.001].

**Table 4:** Odds ratios of logistic regression results predicting mental health and suicide-related outcomes by predictor variables (AAPI sample).

**Outcome variable**	**PORS**	**OMHE**	**PORS × OMHE**	**techfreq**	**Gender**	**Sex**	**Orientation**
Lifetime SI	1.710^***^ [1.388, 2.106]	0.897 [0.747, 1.076]	1.506^***^ [1.313, 1.727]	1.232^*^ [1.047, 1.449]	1.245^**^ [1.090, 1.421]	1.147 [0.864, 1.522]	1.214^***^ [1.129, 1.305]
Past-year SI	1.459 [0.931, 2.287]	1.194 [0.889, 1.603]	1.114 [0.846, 1.467]	1.931^**^ [1.413, 2.639]	1.018 [0.886, 1.169]	0.629^*^ [0.401, 0.986]	1.046 [0.939, 1.165]
Lifetime attempt	1.980^**^ [1.444, 2.715]	1.121 [0.857, 1.468]	1.830^***^ [1.512, 2.214]	1.048 [0.849, 1.294]	1.203^**^ [1.064, 1.359]	1.422 [0.993, 2.034]	1.236^***^ [1.149, 1.329]
Past-year attempt	2.508 [1.107, 5.681]	2.001^*^ [1.299, 3.082]	1.869 [1.166, 2.996]	2.228^*^ [1.385, 3.583]	1.122 [0.927, 1.356]	0.513^*^ [0.224, 1.174]	1.071 [0.905, 1.268]
NSSI	1.808^**^ [1.316, 2.484]	1.299 [1.007, 1.677]	1.602^***^ [1.341, 1.913]	1.229 [1.002, 1.508]	1.288^**^ [1.128, 1.472]	0.802 [0.551, 1.166]	1.182^***^ [1.088, 1.285]

Values are odds ratios (OR).

^*^p < 0.05, ^**^p < 0.01, ^***^p < 0.001.

*P*-values for the interaction terms (PORS × OMHE) were adjusted for multiple comparisons using the FDR procedure within the AAPI sample. PORS, Perceived Online Racism Scale; OMHE, Online Mental Health Information Engagement; PORS × OMHE, Interaction term between perceived online racism and online mental health engagement; techfreq, Daily internet use frequency, measured by how often participants go online for any purpose; gender, Gender identity as self-reported (1 = Man, 2 = Woman, 3 = Other); sex = Sex assigned at birth (1 = Male, 2 = Female, 3 = Intersex); orientation = Sexual orientation (1 = Heterosexual, 2 = Homosexual, 3 = Bisexual, 4 = Other). Outcome variables: Lifetime SI, Ever seriously thought about suicide; Past-year SI, Serious suicidal ideation within the past 12 months; Lifetime attempt, Ever attempted suicide; Past-year attempt, Suicide attempt in the past 12 months; NSSI, Non-suicidal self-injury in the past month. Sample sizes varied across outcomes due to skip patterns; estimates for past-year outcomes are based on smaller subsamples and should be interpreted with caution.

**Table 5:** Coefficients of linear regression results predicting mental health and suicide-related outcomes by predictor variables (AAPI sample).

**Outcome variable**	**PORS**	**OMHE**	**PORS × OMHE**	**techfreq**	**Gender**	**Sex**	**Orientation**
Recent SI (past 2 weeks)	0.338^***^ [0.255, 0.421]	0.299^***^ [0.231, 0.367]	0.147^***^ [0.099, 0.195]	−0.043 [−0.108, 0.021]	0.053 [−0.007, 0.113]	−0.124^*^ [−0.233, −0.015]	0.059^***^ [0.024, 0.093]
Depressive symptoms	0.337^***^ [0.276, 0.398]	0.192^***^ [0.142, 0.242]	0.091^***^ [0.057, 0.124]	0.021 [−0.023, 0.065]	0.047^*^ [0.007, 0.088]	0.057 [−0.023, 0.137]	0.062^***^ [0.038, 0.086]

Values are coefficients (B).

^*^p < 0.05,^**^p < 0.01, ^***^p < 0.001.

*P*-values for the interaction terms (PORS × OMHE) were adjusted for multiple comparisons using the FDR procedure within the AAPI sample. Recent SI, Suicidal ideation in the past 2 weeks; Depressive symptoms, PHQ-9 total score; PORS, Perceived Online Racism Scale; MHE, Online Mental Health Information Engagement; PORS × MHE, Interaction term between perceived online racism and online mental health engagement.

Online mental health engagement was positively associated with past-year suicide attempts [*N* =520, OR = 2.001, 95%CI (1.299, 3.082), *p* = 0.023], NSSI [OR = 1.299, 95%CI (1.007, 1.677), *p* = 0.077)], recent suicidal ideation [*B* = 0.299, 95%CI (0.231, 0.367), *p* < 0.001], and depressive symptoms [*B* = 0.192, 95%CI (0.142, 0.242), *p* < 0.001].

In both groups, sexual orientation consistently emerged as a significant covariate: identifying as a sexual minority was associated with elevated suicidal ideation and depressive symptoms relative to individuals identifying as heterosexual [e.g., Lifetime suicide attempt in Black group: OR = 1.240, 95%CI (1.121, 1.372), *p* < 0.001; AAPI group: OR = 1.214, 95%CI (1.129, 1.305), *p* < 0.001]. Gender identity was also positively associated with suicidality in both groups. Specifically, individuals identifying as women or as gender-diverse (e.g., non-binary or other identities) reported higher levels of suicidality compared to those identifying as men, with this association more pronounced among AAPI participants than Black participants. However, birth-assigned sex showed negative effects only in the Black group [e.g., recent SI: *B* = −0.172, 95%CI (−0.323, −0.021), *p* = 0.025]. In this group, individuals assigned female at birth reported lower levels of recent suicidal ideation compared to those assigned male at birth. Technology use frequency was negatively associated with recent suicidal ideation in the Black group [*B* = −0.070, 95%CI (−0.133, −0.007), *p* = 0.029], but this association was non-significant in the AAPI group [e.g., recent SI: *B* = −0.043, 95%CI (−0.108, 0.021), *p* > 0.05].

### Interaction effects by racial group

#### Black Americans: interaction between online racism and online mental health engagement

Among Black American participants, the latent interaction between online racism and online mental health engagement significantly predicted only one of the seven suicide-related outcomes: recent suicidal ideation (past 2 weeks). The interaction effect was positive and statistically significant [*B* = 0.086, 95%CI (0.027, 0.145), *p* = 0.004], suggesting that the relationship between online racism and suicidal ideation varied as a function of individuals' levels of online mental health engagement.

**Figure 1:**
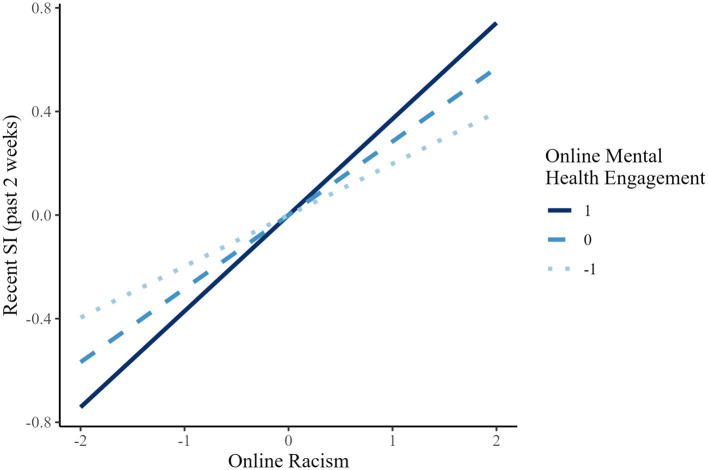
Simple slopes of online racism predicting recent SI (suicidal ideation; past 2 weeks) at different levels of online mental health engagement (Black Americans).

Simple slope analyses revealed that the effect of online racism on Recent suicidal ideation (past 2 weeks) was significant at all levels of mental health engagement (on): low (*B* = 0.198, *p* < 0.001), mean (*B* = 0.284, *p* < 0.001), and high (*B* = 0.371, *p* < 0.001), indicating a consistent pattern of association across low, mean, and high levels of mental health engagement (see Figure 1 for simple slopes depicting the interaction pattern). There were no other significant interaction effects (Tables 2, 3).

#### AAPIs: interaction between online racism and mental health engagement

Among AAPI participants, the latent interaction between online racism and online mental health engagement was statistically significant across multiple suicide-related outcomes. Specifically, significant interactions were observed for past 2 week suicidal ideation [*B* = 0.147, 95%CI (0.099, 0.195), *p* < 0.001], lifetime suicidal ideation [OR = 1.506, 95%CI (1.313, 1.727), *p* < 0.001], lifetime attempt [OR = 1.830, 95%CI (1.512, 2.214), *p* < 0.001], NSSI [OR = 1.602, 95%CI (1.341, 1.913), *p* < 0.001], and depressive symptoms [*B* = 0.091, 95%CI (0.057, 0.124), *p* < 0.001; see Tables 4, 5].

**Figure 2:**
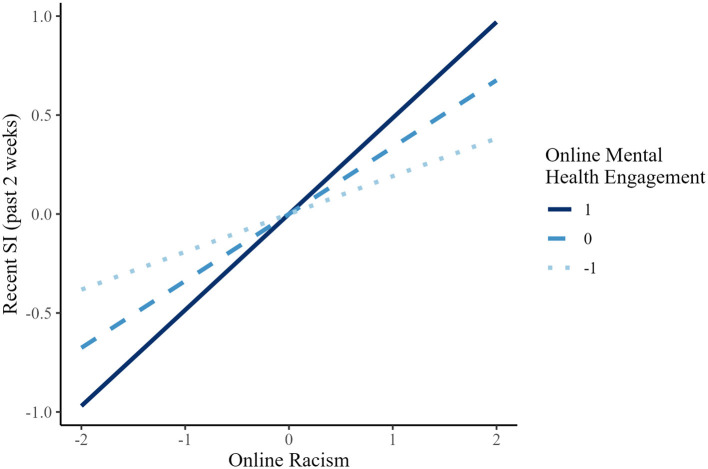
Simple slopes of online racism predicting recent SI (suicidal ideation; past 2 weeks) at different levels of online mental health engagement (AAPI).

**Figure 3:**
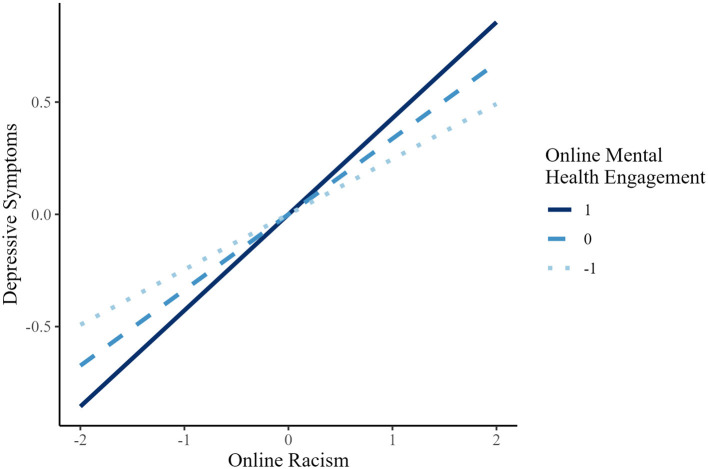
Simple slopes of online racism predicting depressive symptoms at different levels of online mental health engagement (AAPI).

Simple slope analyses (Figure 2) showed that the association of online racism on recent suicidal ideation (past 2 weeks) was statistically significant at low (*B* = 0.191, *p* < 0.001), mean (*B* = 0.338, *p* < 0.001), and high (*B* = 0.485, *p* < 0.001) levels of online MHE. A similar pattern was observed for depressive symptoms (see Figure 3): the association of online racism was significant at low (*B* = 0.246, *p* < 0.001), mean (*B* = 0.337, *p* < 0.001), and high (*B* = 0.428, *p* < 0.001) levels of MHE.

**Figure 4:**
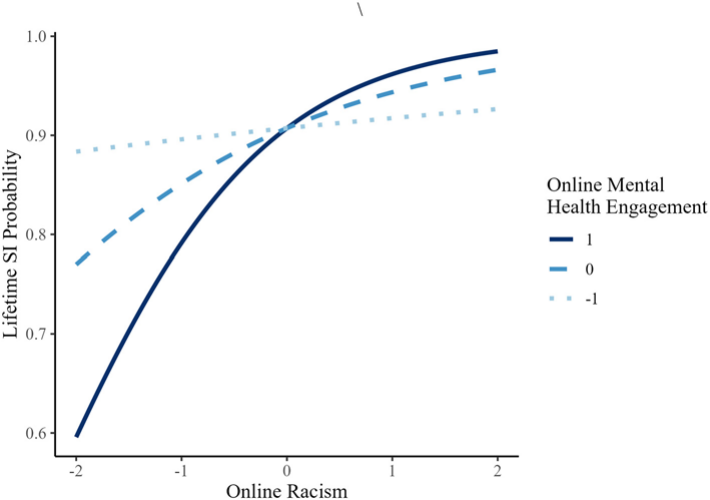
Predicted probability of lifetime SI (suicidal ideation) as a function of online racism at different levels of online mental health engagement (AAPI).

**Figure 5:**
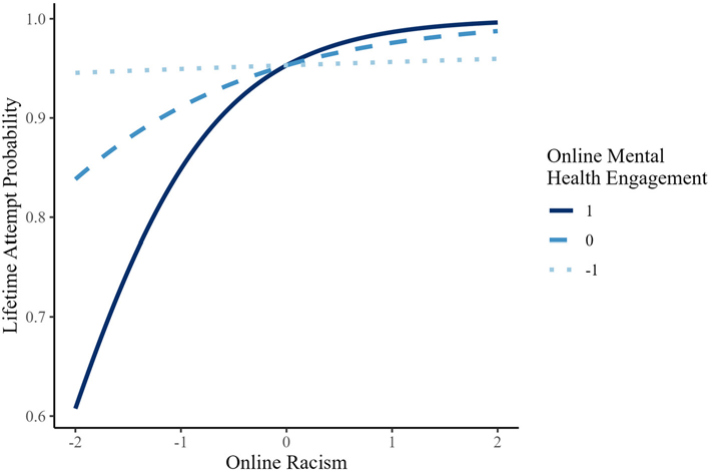
Predicted probability of lifetime attempt as a function of online racism at different levels of online mental health engagement (AAPI).

**Figure 6:**
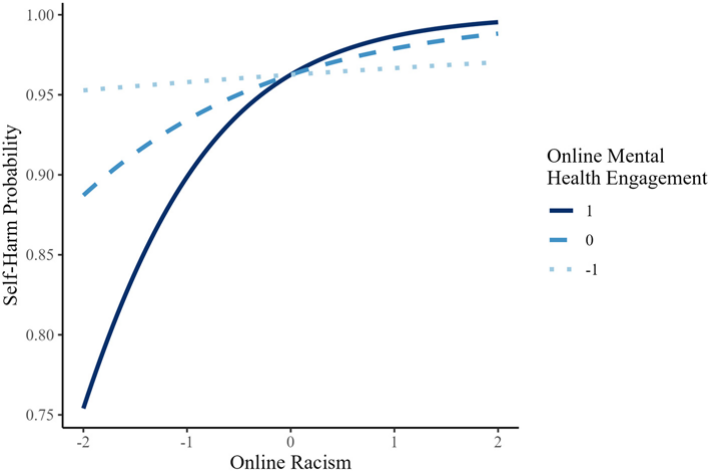
Predicted probability of non-suicidal self-injury as a function of online racism at different levels of online mental health engagement (AAPI).

In contrast, for lifetime suicidal ideation, lifetime attempt, past-year attempt, and NSSI, the association of online racism and these outcomes was not significant at lower levels of MHE, but became statistically significant at mean and high MHE levels (see Figures 4–6 for representative predicted probability patterns across those binary outcomes). For example, in predicting lifetime suicidal ideation, online racism was not significantly associated with suicidal ideation at low MHE (*B* = 0.127, *p* = 0.233, OR = 1.14), but the association became statistically significant at mean (*B* = 0.536, *p* < 0.001, OR = 1.71) and high levels (*B* = 0.946, *p* < 0.001, OR = 2.58). For lifetime suicide attempt, the association was again non-significant at low MHE *(B* = 0.079, *p* = 0.623, OR = 1.08), but became significant at mean (*B* = 0.683, *p* < 0.001, OR = 1.98) and high MHE (*B* = 1.29, *p* < 0.001, OR = 3.63). A similar threshold-dependent pattern was observed for NSSI, the association was non-significant at low MHE (*B* = 0.121, *p* = 0.482, OR = 1.13), and significant at mean (*B* = 0.592, *p* < 0.001, OR = 1.81) and high MHE (*B* = 1.06, *p* < 0.001, OR = 2.88). These results suggest a consistent moderation pattern, whereby the association between online racism and multiple suicide-related behaviors among AAPI emerging adults depends on levels of engagement in online mental health communication, rather than reflecting a linear increase in effect magnitude.

Overall, the results suggest that for AAPI emerging adults, engagement in online mental health discourse delineates the conditions under which online racism is statistically associated with elevated suicide-related risk across multiple domains.

## Discussion

This study aimed to investigate whether online MHE (mental health engagement) moderates the relationship between online racism and depressive symptoms and suicide risk among Black and AAPI emerging adults. Drawing on prior research documenting both benefits and harms of digital mental health resources [e.g., (14, 17)], we conducted an exploratory analysis of their potential protective vs. exacerbating effects. For both groups, we observed patterns in which higher levels of online mental health engagement were associated with a greater likelihood of suicide-related risk in the context of online racism. However, the findings revealed notable group differences. For AAPI emerging adults, higher levels of MHE were associated with a stronger within-group associations between online racism and a broad range of suicide-related outcomes, including suicidal ideation (recent and lifetime), suicide attempts (lifetime), self-harm behaviors, and depressive symptoms. For Black American emerging adults, this pattern was observed primarily for recent suicidal ideation. These results underscore the paradoxical role of MHE in the context of online racism, indicating that online MHE may function as a context-dependent risk correlate rather than a uniformly protective resource, with differing outcomes observed for Black and AAPI groups.

### AAPI emerging adults

One of the main findings of this study is that for AAPI emerging adults, online MHE was not consistently associated with a buffering pattern, but instead was linked to stronger associations between online racism and suicide-related outcomes. While engagement is typically assumed to foster support, a higher frequency of MHE co-occurred with elevated vulnerability in the context of racialized online stress. Several mechanisms may explain this paradox. First, invalidating responses play a key role. When individuals disclose experiences of racism online, they may encounter minimization, subtle victim-blaming, or non-empathetic reactions that retraumatize rather than heal (50). Such invalidation may not only reinforce feelings of isolation but also erode trust in social support systems. In line with this, Yang and Tsai (51) found that actively discussing discrimination online (e.g., messaging friends) was associated with *greater* race-based traumatic stress among AAPI individuals, suggesting that engaging with such content can heighten, rather than relieve, trauma. Second, algorithmic amplification may further exacerbate harm. Engagement with mental health or race-related content can activate algorithmic curation processes that prioritize emotionally charged and polarizing material, potentially increasing exposure to distressing or hostile racial content (52). Such algorithmic echo chambers can immerse users in cycles of distress, rumination, and vicarious trauma, which may undermine the intended benefits of MHE. Third, much of the dominant online mental health discourse remains culturally non-specific. It often emphasizes individual-level “wellness” strategies—such as mindfulness, positive reframing, or cognitive restructuring—to address problems that are fundamentally systemic. For AAPI individuals grappling with racial trauma, these messages can feel invalidating, promote self-blame when such techniques fail, and reinforce isolation (53). Generic, culture-neutral advice may thus disregard collectivist coping styles, such as seeking family harmony or community belonging, that are central to many AAPI cultural frameworks. Taken together, these mechanisms suggest that engagement with online mental health content may expose some individuals to culturally misaligned or emotionally triggering material, a pattern that may help explain the observed associations with heightened suicide-related risk.

### Black American emerging adults

Among Black American emerging adults, the moderating effect of online MHE was evident only for recent suicidal ideation. When exposure to online racism was low, higher MHE was associated with lower levels of suicidal ideation. However, as racist exposure intensified, suicidal ideation among those with high MHE increased sharply, even surpassing those with low engagement—a crossover pattern indicates that the same coping strategy may become maladaptive under severe racial stress.

This shift may reflect a resource depletion process (54), wherein the emotional and cognitive resources gained from online mental health activities are eroded when the platforms providing support also serve as sites of racial hostility (3, 55). For Black emerging adults, whose coping traditions often emphasize communal, spiritual, and collective resilience (56), this contradiction between seeking support and simultaneously encountering racism online may intensify emotional exhaustion and trigger acute stress responses that manifest as short-term spikes in suicidal ideation. However, these episodes may subside as individuals disengage from the online environment or re-engage with offline coping resources (e.g., parents, church, peer networks), preventing the persistence of long-term risk. Compared to AAPI individuals' more disengagement coping strategies (57), Black emerging adults often rely on communal, spiritual, and action-oriented strategies that may heighten immediate emotional responses to online racism yet facilitate recovery through collective support, thus confining the effect to recent rather than long-term suicidality. Moreover, algorithmic exposure again likely contributes to cumulative trauma: Black youth report being funneled by social media algorithms into repeated exposure to racialized violence and hate, fueling ideological fatigue and racial battle fatigue. Together, these findings suggest that under conditions of pervasive online racism, online mental health engagement may be insufficient to offset racialized stress and, in some contexts, may coincide with heightened vulnerability rather than protection.

This paradoxical pattern carries important theoretical implications. It supports a context-dependent coping framework, suggesting that the effects of MHE depend on the sociocultural and stress context rather than being uniformly adaptive or maladaptive (58, 59). More specifically, the findings align closely with the Differential Susceptibility to Media Effects Model [DSMM; (60, 61)], which posits that media effects are conditional on individual susceptibility factors and contextual inputs rather than uniformly experienced. Within this framework, online mental health engagement can be conceptualized as a susceptibility factor that heightens responsiveness to digital environments. In supportive digital and social environments, such engagement may be associated with enhanced emotion regulation and resilience, but under hostile or invalidating conditions, such as exposure to racism or cultural invalidation, it may be linked to greater distress and trauma (51, 53). Integrating DSMM with Carter's (62) Race-Based Traumatic Stress Model, 51 Conservation of Resources Theory, and emerging perspectives in digital media psychology (52) allows us to articulate what we term a Digital Susceptibility Hypothesis. This hypothesis posits that online mental health engagement is not inherently protective but reflects differential susceptibility to digital environments, such that engagement intensifies the psychological impact, beneficial or harmful, of online racialized experiences. By situating this hypothesis within DSMM, the present study extends existing media-effects theory into the domain of racialized digital stress and suicide risk. By highlighting these intersecting patterns, this study extends racial trauma and digital mental health research and offers a conceptual bridge for understanding how racialized stress interacts with digital coping in the modern era. These findings point to the need for digital mental health interventions that go beyond individual symptom management to address structural racism within online spaces—such as through culturally tailored content moderation, representation in algorithmic design, and the inclusion of Black community-based healing models.

## Limitations

This study has several limitations. First, the cross-sectional design precludes causal inference regarding the directionality of the observed associations. Although systematic moderation patterns were identified, the temporal ordering of online racism exposure, online mental health engagement, and suicide-related outcomes cannot be established. It remains unclear whether exposure to online racism contributes to heightened suicide-related risk among individuals highly engaged in online mental health content, or whether individuals experiencing greater distress are more likely to engage in online spaces where exposure to racism is also more frequent.

Second, several suicide-related outcomes were assessed using dichotomous indicators (e.g., lifetime and past-year suicidal ideation and attempts), which may be subject to lower reliability and reduced sensitivity compared to continuous measures. Future research would benefit from incorporating multi-item or continuous measures of suicidality to more precisely capture severity and temporal dynamics.

Third, the analyses did not account for broader structural and contextual factors that may shape both online mental health engagement and suicide-related vulnerability. Socioeconomic stressors, offline experiences of discrimination, and patterns of media exposure may jointly influence individuals' likelihood of engaging in online mental health spaces and their exposure to racialized stress, limiting the ability to isolate independent pathways in the present data. Relatedly, although the broader survey included validated measures of mental health stigma, help-seeking attitudes, and perceived barriers to care, these constructs were not incorporated into the current analytic models in order to maintain focus on the latent interaction framework. It is therefore possible that stigma-related disengagement from mental health discourse or reduced help-seeking may partly account for the elevated suicide risk observed among individuals reporting low levels of online mental health engagement.

Fourth, online mental health engagement was modeled as a single latent construct, although it likely encompasses heterogeneous behaviors (e.g., information seeking, emotional disclosure, peer support) with potentially distinct implications for mental health. More fine-grained measurement is needed to clarify which forms of engagement confer risk vs. resilience. Furthermore, the use of an online panel data (non-probability) may limit generalizability, as participation required internet access and voluntary engagement with online surveys.

Fifth, both “AAPI” and “Black American” represent broad and internally diverse population categories, and within-group differences in ethnicity, culture, and lived experiences may shape how individuals experience online racism and engage with digital mental health resources. Accordingly, the findings should be interpreted as reflecting potential general patterns rather than uniform processes across all subgroup populations.

Sixth, the low survey completion rate (17.6%) from removing cases without 100% survey completion introduces the possibility of non-response bias, as the participants who completed the protocol may differ systematically from those who did not. For instance, the length and sensitive nature of the survey—which inquired deeply into experiences of racial victimization and suicide ideation—may have led to a “survivor bias,” where individuals with higher psychological resilience or those less acutely triggered by the content were more likely to reach the end. It is also possible that the sample is biased toward those with a particularly strong motivation to report these experiences, potentially over-representing the severity of the associations found. Furthermore, the high dropout rate may reflect general survey fatigue common. Despite these potential biases, the final sample of 2,800 provides significant statistical power for the complex moderation analyses conducted. However, the findings should be viewed and interpreted with these limitations in mind.

## Implications for future research

Future research should differentiate the forms and motivations of online MHE. Passive exposure may exacerbate helplessness and rumination, whereas active and interactive engagement may enhance emotional regulation and social connectedness. Fine-grained measurement and computational modeling could help illuminate these dynamics. Researchers should also improve the ecological validity and representativeness of their samples by integrating data from multiple recruitment sources—including social media platforms, community-based samples, and digital behavioral data—to capture more diverse online experiences. Finally, it will be crucial to disaggregate racial and ethnic subgroups within broad categories. Accounting for differences in ethnicity, immigration generation, and cultural background can clarify which populations are most at risk and inform the development of culturally grounded and intersectionality informed suicide prevention strategies. Moreover, future studies may replicate our findings while taking into consideration internalized racism as a potential mediator through which online racial/ethnic discrimination may be linked to suicide ideation, with ethnic identity commitment serving as a potential protective factor against these harmful effects.

## Conclusion

This study showed a paradoxical relationship between online mental health engagement, online racism, and suicidal thoughts and behaviors among Black and AAPI emerging adults, illustrating that digital coping is not uniformly protective across contexts. While extant literature suggests that online mental health engagement would buffer the mental health impact of racial stress, our findings indicate that under conditions of heightened online racism, online mental health engagement may be associated with greater risk for depression/suicidal thoughts/behaviors. For AAPI emerging adults, greater online mental health engagement intensified associations between online racism and depressive and suicide-related outcomes. Among Black emerging adults, the intensified association was only evident for recent suicidal ideation. Together, these findings support a context-dependent coping framework that accounts for the possibility that the effects of online mental health engagement can vary with the surrounding sociocultural and stress environment, such that online mental health engagement can both heal and harm.

## Data Availability

The raw data supporting the conclusions of this article will be made available by the authors, without undue reservation.
